# The U.S. Tax Program for Swiss banks: what determined the penalties?

**DOI:** 10.1186/s41937-018-0024-0

**Published:** 2018-12-17

**Authors:** Yvan Lengwiler, Albana Saljihaj

**Affiliations:** 1 University of Basel, Faculty of Business and Economics (WWZ) and Center for Innovative Finance (CIF), Basel, CH-4002 Switzerland; 2 University of Basel, Faculty of Business and Economics (WWZ), Basel, CH-4002 Switzerland

**Keywords:** Tax evasion, Bank secrecy, G21, H26

## Abstract

The U.S. Tax Program for Swiss banks is a very significant part of the recent history of the Swiss financial industry. It has accelerated the transformation of the Swiss banking industry from a system that relied on bank secrecy to a much more compliant one. It was also rather costly for the banks involved. This short paper tries to identify the determinants of the individual penalties that were levied by the DoJ. We find that U.S. assets under management is the most important determinant. However, the average size of the accounts, the behavior of the bank vis-à-vis its American clients, the solvency of the bank, and the point in time when the bank settled with the DoJ also matter.

## Introduction

In August 2013, the U.S. Department of Justice (DoJ) and the Swiss Federal Department of Finance issued a joint statement that created the *U.S. Tax Program for Swiss Banks* (“the program”). This program paved the way for ending the conflict that had emerged between the two countries concerning Swiss bank secrecy (DoJ 2013).

At the time, the DoJ had collected evidence that several Swiss banks had systematically helped U.S. tax subjects to evade taxation. Several Swiss banks were already under criminal investigation by the DoJ. The largest of these banks, UBS, had settled in 2009 and received a deferred prosecution agreement (DPA) in exchange for full cooperation with the DoJ, including providing names of clients and employees, and a penalty of USD 780 million. One bank (Wegelin) collapsed in 2012 as a consequence of the conflict with the DoJ. Six major banks (Credit Suisse, Bank Julius Bär, Pictet, HSBC, Zürcher Kantonalbank, and Basler Kantonalbank) and eight smaller banks had also been charged but had not yet settled at the time of the creation of the program. Other banks were at risk of being implicated in the DoJ’s investigation. The whole Swiss banking industry appeared to be under siege.

International pressure on Swiss bank secrecy had already been mounting for some time. Before the U.S. came into play, there had been a series of initiatives led by the OECD to increase transparency and induce cooperation among countries on taxation. With respect to bank secrecy, the OECD tried to establish the free exchange of information between tax authorities, or ideally the automatic exchange of information on tax subjects. This amounts to abolishing bank secrecy for tax purposes. The strategy of the OECD relied mainly on blacklisting non-cooperative jurisdictions, thereby hoping that this public shaming would prove effective. In successive negotiations between Switzerland and the EU, a withholding tax on interest income was introduced, and the distinction between tax evasion and tax fraud was removed, thereby easing the way for an EU tax authority to request information on bank accounts in Switzerland about which they were suspicious. However, in all these negotiations, bank secrecy itself was fully preserved.

Delaloye et al. (2012) use this period of prolonged negotiations to estimate the value of bank secrecy for UBS and CS (the two largest international universal banks in Switzerland) and two large Swiss private banks (Julius Bär and Vontobel). They extract the effect on the stock prices of these banks that is due to particular events connected to the negotiations. The only event that has a negative effect on UBS and CS was a letter by EU Commissioner Frits Bolkestein published in the Financial Times. The authors interpret this letter as hinting towards possible sanctions and loss of access to the EU financial market. Two other events related to the introduction of the withholding tax affected the two private banks, but not the large universal banks. The authors conclude that bank secrecy was of significant value only for the two private banks, whereas its value for the two international universal banks was zero. These banks feared loss of market access much more and might therefore have been in favor of abolishing bank secrecy in order not to jeopardize market access.

Emmenegger (2017) asks the question of why bank secrecy, having been well established for decades in Switzerland, ultimately fell. He argues essentially that the Swiss state depends on the viability of the large international banks, and these banks depend on access to the U.S. financial market. By threatening to deny these banks access to this important market, the USA was able to force the Swiss government to agree. Having publicly admitted to wrongdoing, Switzerland was then unable to withstand reform, also vis-à-vis other jurisdictions.

We do not find this argument completely convincing. It is obvious that access to the U.S. financial market is vital, but it is not obvious that access to the EU financial market is less vital. So why was the U.S. so much more successful than the EU in its handling of Switzerland? We are not legal or political science scholars, but it is interesting that the EU was negotiating with the Swiss government, whereas the DoJ was attacking Swiss corporations. Its first target was the biggest bank in the country, no less. This is in line with the guidelines set by the DoJ with respect to charging corporations: “[…] corporations are likely to take immediate remedial steps when one is indicted for criminal conduct that is pervasive throughout a particular industry, and thus an indictment often provides a unique opportunity for deterrence on a massive scale […]” (Holder (1999), Section I.B). It is conceivable that this strategy was far more effective than the shaming and negotiating strategy of the OECD and the EU. We agree with Emmenegger (2017) about the aftermath of the program: after it was publicly established that Swiss banks had helped customers to avoid taxation, it was impossible to keep bank secrecy alive.

The program is a significant part of recent Swiss financial history. It contains material for scholars of law, political scientists, historians, and, of course, economists. The aim of this short paper, however, is not to analyze the long-term effects of the program, but is much more mundane. We simply study the determinants of the penalties that were paid by category 2 banks. We find that there are five determinants. First, the most important variable determining the fines is the maximum U.S.-related assets under management. This finding is in line with the formal definition of the program. Second, the DoJ lists particular types of behavior it found in the participating banks. We find a few such behaviors that prove valuable in explaining the penalties. Third, the average assets under management per account is also an important determinant. Banks with the same assets under management but whose assets belonged to a larger number of smaller clients paid larger penalties than if the bank had fewer, but larger, clients. Fourth, we find some evidence that the solvency of the banks at the end of 2013 may have played a role as well. Banks that had a low leverage ratio (i.e., high levels of capital compared to their balance sheets) received higher penalties than banks that were less solvent. Fifth, the DoJ seems to have eased the fines as the program evolved. Controlling for the other variables, we find that the fines for the banks that settled last were significantly smaller than the ones that settled early. We have no indication why this is the case.

## Basics of the program

Participation in the program was voluntary, and some banks indeed opted not to participate. The program allowed the industry to resolve the U.S. tax issue in a structured and relatively predictable manner. The last paragraph of the DoJ Tax Division’s comment on the program makes this very clear:

“Each eligible Swiss bank should carefully weigh the benefits of coming forward, and the risks of not taking this opportunity to be fully forthcoming. A bank that has engaged in or facilitated U.S. tax-related or monetary transaction crimes has a unique opportunity to resolve its criminal liability under the Program. Those that have criminal exposure but fail to come forward or participate but are not fully forthcoming do so at considerable risk”. (DoJ Tax Division (2013), p. 4)

The first step for a participating bank is to request permission from the Swiss Federal Department of Finance to participate. Such permission is necessary because cooperating with a foreign government to the extent required by the program without prior permission constitutes a breach of Art. 271 Abs. 1 of the Swiss penal code.^1^ In July 2013, the Finance Department published a model request for banks to apply for such permission (EFD 2013).

The second step is for the banks to declare their willingness to participate in the program by submitting a *letter of intent* (LOI). The deadline for this was December 31, 2013. The program divided the banks into four categories, and banks have to indicate to which category they want to belong.^2^ Category 4 banks are financial institutions with local client bases who are deemed compliant. Category 3 banks are banks that are not category 4, but who have not committed tax-related or monetary transactions offenses against U.S. law. Category 2 is for banks that are not category 1 but have reason to believe they may have committed tax-related offenses. Category 1 are banks that were already under criminal investigation (the aforementioned fourteen banks). These banks are excluded from the program.

Category 3 and 4 banks have to provide evidence for their classification, which is then verified by an independent examiner (at the expense of the bank). In exchange, the bank is offered a non target letter (NTL), which is essentially just a document stating that the bank is not, at that time, the target of any criminal investigation authorized by the DoJ’s tax division.

Category 2 banks are the focus of the program. Banks that fulfill the requirements of the program are offered a non-prosecution agreement (NPA). An NPA is very similar to a DPA. In both cases, the bank is not convicted of any crime and does not formally admit guilt, but full cooperation and typically the payment of a penalty are required.^3^ The process and the obligations of category 2 banks are explained in the next section in greater detail.

All category 2, 3, and 4 banks have in the meantime settled with the DoJ and have received NPAs or NTLs. Some category 1 banks, however, have not yet settled. Two of these banks have been liquidated in the meantime (Bank Frey and Neue Zürcher Bank). According to DoJ data, the category 1 banks that have settled so far had an aggregate maximum amount of assets under management (AuM) belonging to U.S. clients of USD 38 billion. They have paid penalties of about USD 4.4 billion. The category 2 banks had in total maximum assets under management belonging to U.S. clients of roughly USD 50 billion and have, in sum, paid penalties of USD 1.37bn.^4^ Other Swiss (non-bank) companies are also under investigation or have been charged and have settled similar disputes with the DoJ. For instance, the wealth management company Prime Partners in Geneva is a family office, not a bank. They received an NPA in exchange for full cooperation (e.g., naming of clients) and a penalty of USD 5 million.^5^ Since Prime Partners is not a bank, the program was not open to them and this paper does not include such cases.

## Category 2

In order for a bank to be granted an NPA, it has to fulfill three obligations. It must provide detailed *information*, it must provide *assistance* to the DoJ in implicating other individuals, and it must pay a *penalty*. The process is explained in detail in DoJ (2013) and DoJ Tax Division (2013).

**Information** The bank must disclose the total number of U.S.-related accounts, as well as the maximum balance on these accounts for three periods: on August 1, 2008, between August 1, 2008, and February 28, 2009, and after February 28, 2009.

In addition, the bank must provide information about the amount and form of the transfer of funds into and out of the account during the applicable period. In particular, it must show where the funds came from and where they went to (i.e., identification of intermediary or financial institution and its domicile). This information is clearly intended so that the DoJ is able to follow the money and implicate other institutions or individuals.

The bank must identify further its internal procedures for the handling of U.S. clients and accounts. This information includes the names of employees involved in acquiring, operating, and supervising such accounts. In particular the names of the relationship manager, client advisor, asset manager, financial advisor, trustee, fiduciary, nominee, attorney, or accountant at any time during the relevant period have to be submitted. The information also includes internal reporting and communication with management on such matters.

The above information has to be verified (at the expense of the bank) by an independent examiner.

**Assistance** Interestingly, the bank does not disclose the identities of the U.S. account holders themselves. Doing so would violate Swiss bank secrecy law. However, upon request, the bank will provide testimony and assist the USA in any criminal proceedings. In particular, the bank provides all information necessary for the USA to draft treaty requests to seek account information.

Furthermore, the banks will close all non-compliant U.S.-related accounts. They will also prevent their employees from assisting U.S. account holders with further concealment. Moreover, the banks will open new U.S.-related accounts only if they can ensure that the account will be declared to the USA and will be subject to disclosure by the Swiss bank. This effectively terminates Swiss bank secrecy for U.S. customers vis-à-vis the U.S. government for category 2 banks.

**Penalty** The program specifies a formula for computing the penalty:

“Upon execution of an NPA, the Swiss Bank will agree to pay as a penalty:

1. for U.S. Related Accounts that existed on August 1, 2008, an amount equal to 20% of the maximum aggregate dollar value of all such accounts during the Applicable Period;
2. for U.S. Related Accounts that were opened between August 1, 2008, and February 28, 2009, an amount equal to 30% of the maximum aggregate dollar value of all such accounts; and
3. for U.S. Related Accounts that were opened after February 28, 2009, an amount equal to 50% of the maximum aggregate value of all such accounts.

The determination of the maximum dollar value of the aggregated U.S. Related Accounts may be reduced by the dollar value of each account as to which the Swiss Bank demonstrates, to the satisfaction of the Tax Division, was not an undeclared account, was disclosed by the Swiss Bank to the U.S. Internal Revenue Service, or was disclosed to the U.S. Internal Revenue Service through an announced Offshore Voluntary Disclosure Program or Initiative following notification by the Swiss Bank of such a program or initiative and prior to the execution of the NPA”. (DoJ 2013, section II.H)

Note that the penalties are rather steep. They are between 20 and 50% of the maximum aggregate amount that was in these accounts. This is certainly more than the profit the banks made with these accounts, and very likely more than the tax that was evaded. Of course, not all U.S. accounts were undeclared, and it is in fact likely that many U.S. clients were tax compliant. However, the burden of proof that some of these accounts were in fact declared to the IRS lies with the banks.

Even though the program prescribes a rigid formula for the penalty, the DoJ clearly had some degree of freedom in determining the fines. On the Tax Program website, they state: “It is indeed essential to remember that banks receive a rebate if, for example, they managed to push clients into the IRS voluntary disclosure program. *Furthermore, extraordinary cooperation might play a role in reducing the fine as well.* It follows that a low penalty could be an indication that the bank had very little undeclared U.S.-related assets or that the institution closed undeclared accounts early in the time period of the U.S. Tax Program, or that a lot of its U.S. clients participate in a voluntary disclosure program initiated by the bank, *or even that the bank strongly cooperated with the DoJ.*” (see http://www.ustaxprogram.com/penalty-statistics/, emphases added).

## Data

Initially, 87 banks were in category 2. Nine of those^6^ subsequently left the program or switched to a higher category, so 78 banks remained in category 2 and went through with the process. The DoJ provides the individual NPAs and summary data for all the 78 banks that remained in category 2 on the website http://www.ustaxprogram.com/banks/ (see also https://www.justice.gov/tax/swiss-bank-program).

According to the program as described in the previous section, the penalty is a function of the maximum amount of assets under management (AuM) in three different periods, minus some potential rebate for extraordinary cooperation. This hypothesis cannot be tested, however, because the DoJ has not published the AuM for the individual banks and the three periods separately. For most participating banks, they only publish the maximum AuM over all three periods together.

The DoJ has also published the maximum number of U.S.-related accounts for each bank. According to the program, this data should not affect the penalty. Interestingly, the DoJ was careful to document this information for each bank. This leads us to believe that it did play a role in some way. We will argue that the average assets per account, AuM/accounts, is a useful explanatory variable.

The NPAs describe in some detail the DoJ’s findings concerning the activities of each bank relevant to allowing or helping customers avoid taxation. We have collected 30 such practices, see Table 1. The practices range from rather benign behavior, such as “e-banking, retail and private banking services for U.S. clients” (A24), to much more dubious actions, such as “assisted in the falsification of documents” (A3). We will experiment with different collections of these activities as regressors to find a combination that helps explain the penalties.

**Table 1:** Activities of banks as described by the DoJ

ACTIVITIES			
*A1 [75]*	*Post withholding service*	A16 [19]	Assisting the bank’s clients in concealing assets and income from IRS
A2 [61]	Numbered accounts/pseudonyms/code name	A17 [20]	maintaining insurance wrappers
*A3 [3]*	*Assisted in the falsification of documents*	A18 [39]	(Account closure via) excessive cash, precious metal withdrawal, cashing checks, bearer shares, or fictitious donations
A4 [37]	Cash cards or credit cards (Anonymous cash withdrawal)	A19 [29]	Transfer of assets from closed accounts to non-US-related accounts or accounts at other banks held by non-US relatives/friends or removing name of US taxpayer clients from joint accounts
*A5 [2]*	*Lombard loans secure by (uncleared) assets*	A20 [18]	Permitted relationship managers to meet or have direct contact with US beneficial owner, even such who did not have powers of attorney over the entity accounts
A6 [45]	Inaccurate account documentation (missing or false forms)	A21 [15]	Referred or provided US taxpayers with the names of outside services providers who could create structures or assisted them with creating structures
A7 [22]	Accounts in the name of non-US-persons that were owned by US taxpayers	*A22 [1]*	*Remove bank letterhead from account statements*
A8 [56]	Accounts in the name of foundations/companies with US residents as their beneficial owners	A23 [5]	Transmitting undeclared assets to a US taxpayer client in a hidden manner (for instance by delivering cash in person)
A9 [4]	Advise clients to conceal their US nexuses from bank documentation or avoid bringing account information to the US	A24 [9]	e-banking, retail, and private banking services for US clients
A10 [9]	Issuing checks drawn on the client’s/customer’s account or wire transfers	A25 [6]	Transfer of assets from US-related accounts through non-US accounts en route to accounts at unaffiliated banks to conceal the US relatedness of these accounts
*A11 [3]*	*Issuing checks drawn on one of the bank’s accounts*	*A26 [2]*	*Discussing Swiss banking secrecy with US taxpayer clients*
A12 [45]	Opening accounts for persons that left other banking being investigated by the DOJ or have been exited or left during the financial crisis	*A27 [1]*	*Failing to adopt an account-closing protocol*
*A13 [1]*	*Destroying correspondence upon request*	*A28 [1]*	*Transitory account*
A14 [10]	Concealment of communications through prepaid mobile phones, fax, or personal email or communication by confidential means in general	*A29 [1]*	*Advisory or booking center*
A15 [5]	No registration of US taxpayer clients as US persons in the bank’s IT system	A30 [24]	Structured payments

Note: Ax is the code used in the regression tables. The number in square parentheses is the number of banks that are assigned the respective activity in the DoJ findings. Activities written in italic font are not used in regressions because they are too common or too rare

Finally, we have collected balance sheet data for as many banks as possible for the year 2013. In particular, we have collected the size of the balance sheets, capital, earnings, and profit of 60 banks. For 18 banks—all specializing in private banking^7^—we do not have access to annual reports.

Table 2 provides descriptive statistics of the data. The penalty, assets, number of accounts, and average account size data are extremely skewed. The logarithmic versions of those variables are more symmetrically distributed. In order to avoid the results being dominated by the banks with the largest penalties, most AuM, and most or largest accounts, we will mostly work with logarithmic data. One bank did not have to pay a penalty (Banca Intermobiliare di Investimenti e Gestioni (Suisse) SA), so this bank is dropped from our sample as well.

**Table 2:** Some descriptive statistics (penalties and AuM in million USD; capital and balance sheet in million CHF)

	Penalty	AuM	Accounts	AuM per account	Capital 2013	Balance sheet 2013	Capital share 2013
Variable							
Mean	17.53	641.33	449.90	1.504	851.17	10622.4	0.1056
Median	4.202	200.48	187	1.112	395.32	4336.64	0.08323
Maximum	211.0	7650.0	3500	7.914	6947.0	115193	0.3884
Minimum	0.00	6.90	13	0.085	13.686	81.125	0.01277
Std dev	36.88	1209.6	649.7	1.450	1180.8	17261.7	0.0711
Skewness	3.594	3.649	2.866	2.260	2.752	3.973	2.511
Kurtosis	16.888	18.416	11.843	9.123	13.275	23.626	9.611
Observations	78	78	78	78	60	60	60
Log(variable)							
Mean	1.481	5.386	5.375	0.0106	5.772	8.203	– 2.431
Median	1.448	5.313	5.231	0.0822	5.980	8.378	– 2.500
Maximum	5.352	8.942	8.161	2.069	8.846	11.654	– 0.9458
Minimum	– 4.701	1.932	2.565	– 2.468	2.616	4.396	– 4.361
Std dev	1.844	1.516	1.272	0.9394	1.615	1.683	0.5416
Skewness	– 0.479	0.057	– 0.056	– 0.304	– 0.215	– 0.269	– 0.539
Kurtosis	4.114	2.793	2.665	3.062	1.880	2.241	6.980
Observations	77	77	77	77	59	59	59

**Fig. 1:**
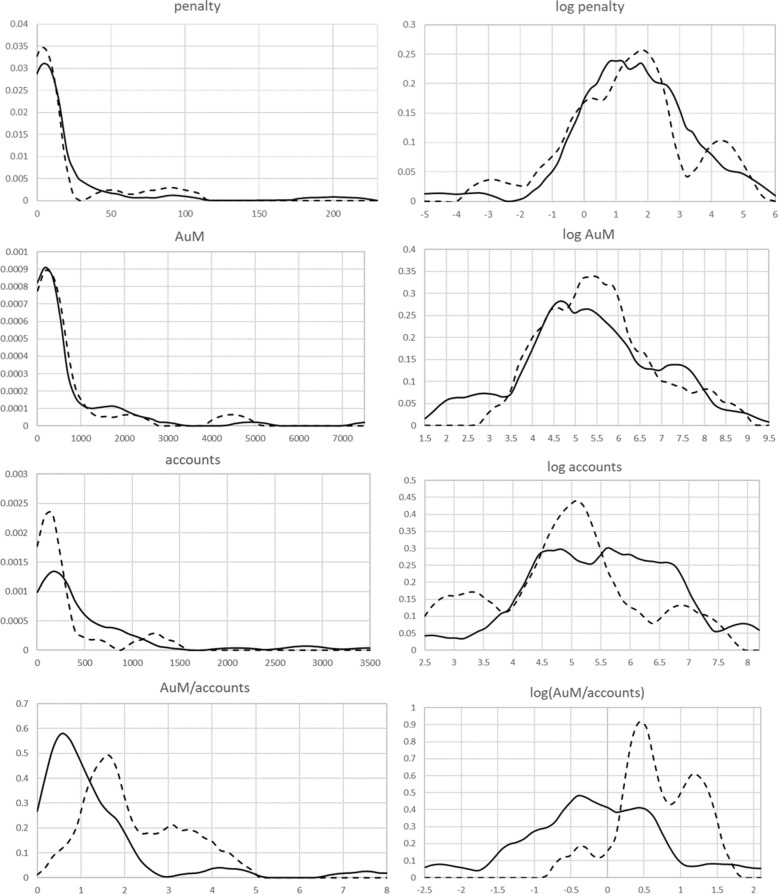
Distributions (kernels) of some data. The solid lines are for the 60 banks for which we have balance sheet data; the dashed lines are for the 18 banks for which we do not

Figure 1 depicts more detailed information about the data in order to assess selection effects when dropping the private banks for which no balance sheet data are available. The chart provides kernel estimates of the main variables for the subset of banks that are in the sample with balance sheet data, and the ones that are excluded from this sample. Visual inspection suggests that there is not a great difference between the two samples, except with respect to average account size. The excluded private banks had significantly larger accounts than the other banks.

## Results

As stated in the program, the determinants of the penalty are the maximum aggregate assets under management in U.S.-related accounts. The difficulty is that the program distinguishes between three points in time, namely August 1, 2008, between August 1, 2008, and February 28, 2009, and after February 28, 2009. However, the data published by the DoJ for most participating banks do not distinguish these three periods; only the maximum over the three periods is published. Moreover, the penalty is levied only on those assets that were unknown to the IRS at the time the program began, but the published data does not reveal the share of assets that were shown by the bank to be tax compliant. As a consequence, our model is necessarily miss-specified.

Category 2 banks held, according to the DoJ data, about USD 50 billion U.S. assets under management (AuM). If all these assets were not tax compliant, the total penalty would have to be between 20 and 50% of this value, that is, between USD 10 and USD 25 billion. Yet, the penalties were only USD 1.37 billion, which is between 5.5 and 13.7% of the values we would expect if all AuM were non-compliant. Therefore, apparently, the banks were unable to establish tax compliance for 5.5 to 13.7% of their AuM, or vice versa, between 86.3 and 94.5% of the assets in category 2 banks were indeed tax compliant.

Despite the unavoidable miss-specification, a regression

1 $$ p_{i} = \beta_{0} + \beta_{1}\cdot\text{AuM}_{i} + \varepsilon_{i},   $$

where *p*_*i*_ is bank *i*’s penalty, is a start. If the complete AuM had evaded U.S. taxation, *β*_*i*_ should be between 0.2 and 0.5, according to the definition of the program. In Table 3, column 1, we report that this coefficient is only 0.02, which is consistent with the assessment we made before that the major part of the AuM were indeed tax compliant.

**Table 3:** Regression results

**Specifications**	**(1)**	**(2)**	**(3)**	**(4)**	**(5)**	**(6)**	**(7)**
AuM	+ 0.0207**						
Log(AuM)		+ 0.9417****	+ 0.7713****	+ 0.3279**	+ 1.0145****	+ 1.0682****	+ 1.0433****
A4			+ 0.5595**	+ 0.5929****	+ 0.5929****	+ 0.5388****	+ 0.5786****
A9			+ 0.9724****	+ 0.8040****	+ 0.8040****	+ 0.6156****	+ 0.7050***
A19			+ 0.6060***	+ 0.5422****	+ 0.5422****	+ 0.3094*	+ 0.3290
Log(accounts)				+ 0.6866****			
Log(AuM/actounts)					– 0.6866****	– 0.7780****	– 0.7726****
log(AuM/accounts)^2^						– 0.2748***	– 0.2560**
Const	+ 4.2425	– 3.5912****	– 3.2208****	– 4.5066****	– 4.5066****	– 4.4321****	– 4.3836****
Nobs	78	77	77	77	77	77	59
Std endogenous	36.8802	1.8438	1.8438	1.8438	1.8438	1.8438	1.8413
Std regression	27.2337	1.1741	1.1096	0.9695	0.9695	0.9186	0.9148
R^2^ adjusted	0.4547	0.5945	0.6378	0.7235	0.7235	0.7518	0.7531
**Specification**	**(8)**	**(9)**	**(10)**	**(11)**	**(12)**	**(13)**	**(14)**
Log(AuM)	+ 1.2010****	+ 1.0194****	+ 1.0539****	+ 1.0972****	+ 1.1079****	+ 1.1554****	+ 1.2004****
A4	+ 0.5144**	+ 0.5891****	+ 0.5940****	+ 0.5629****	+ 0.5689****	+ 0.5339****	+ 0.5310****
A9	+ 0.6398**	+ 0.7111***	+ 0.6129**	+ 0.6343**	+ 0.5775**	+ 0.6045**	+ 0.4996**
A19	+ 0.2734	+ 0.3372	+ 0.3205	+ 0.2726	+ 0.3779	+ 0.3199	
Log(AuM/actounts)	– 0.9800****	– 0.7417****	– 0.7971****	– 0.8263****	– 0.8060****	– 0.8393****	– 0.8706****
Log(Au M/accounts)^2^	– 0.2698**	– 0.2532**	– 0.2403**	– 0.2388*	– 0.2519**	– 0.2497**	– 0.2876***
Log(balance sheet)	– 0.1338						
Log(capital)		+ 0.0207					
Logfcapital/balance sheet)			+ 0.4797*		+ 0.5616*		
Capital/balance sheet				+ 3.7302*		+ 4.3191**	+ 4.5261***
Date of NPA					– 1.3248**	– 1.2631**	– 1.2138**
Const	– 4.0999****	– 4.3804****	– 3.2922****	– 5.0435****	– 2.7464****	– 4.8050****	– 4.9399****
Nobs	59	59	59	59	59	59	59
std endogenous	1.8413	1.8413	1.8413	1.8413	1.8413	1.8413	1.8413
std regression	0.9137	0.9236	0.8822	0.8921	0.8420	0.8567	0.8601
R^2^ adjusted	0.7537	0.7484	0.7704	0.7653	0.7909	0.7835	0.7818

Note: *,**,***,**** indicates significance at the 10, 5, 2, and 1% level, respectively, using White standard errors. Coefficients of log(AuM) that are printed in italics are statistically significantly different from unity (at 5% level). The dependent variable in specification (1) is penalty, in (2) to (14) the variable is log (penalty)

To account for the highly skewed distribution of the data, we will use the logarithmic specification of (1). Note that now the coefficient *β*_1_ should be 1 according to the definition of the program because the relationship between AuM and penalty ought to be linear, hence exhibiting unit elasticity. Table 3, column 2, reports that *β*_1_ is indeed close to unity, though statistically slightly smaller.^8^

We now add the activities, as identified by the DoJ in the individual NPAs, as explanatory variables. If we add these activity dummies individually to Eq. (2), activities A4, A9, A19, A20, and A24 turn out to be significant at the 5% level. If we use these five dummies simultaneously, A20 and A24 lose their significance. We therefore keep the remaining three activities in the regression,

3 $$\begin{array}{*{20}l} {} \log(p_{i}) &= \beta_{0} + \beta_{1}\cdot\log(\text{AuM}_{i})\\ &\quad+ \beta_{2}\cdot\text{A4} + \beta_{3}\cdot\text{A9} + \beta_{4}\cdot\text{A19} + \varepsilon_{i}.  \end{array} $$

The results are reported in Table 3, column 3. The coefficients of the activities are all positive, indicating that banks that did pursue these activities did face larger fines.

As reported, the DoJ carefully collected and reported the number of U.S.-related accounts, despite the fact that the number of accounts is not a determinant of the penalty according to the definition of the program. However, the (logarithmic) number of accounts indeed turns out to have a statistically significant explanatory power,

4 $$\begin{array}{*{20}l} \log(p_{i}) &= {\beta_{0}} + {\beta_{1}}\cdot\log(\text{AuM}_{i}) \\ &\quad+ {\beta_{2}}\cdot\text{A4} + {\beta_{3}}\cdot\text{A9} + {\beta_{4}}\cdot\text{A19} \\&\quad+ {\beta_{5}}\cdot\log(\text{accounts}) +{\varepsilon_{i}}.\qquad\qquad  \end{array} $$

Table 3, column 4, reveals one unsatisfactory feature of this specification, though the elasticity of AuM with respect to penalties is clearly not 1 anymore. According to the program, there should be a linear relationship between these variables, and thus the elasticity should be unity. We can remedy this shortcoming by not using the number of accounts per se as a regressor, but the average size of the accounts, AuM/accounts,

5 $$\begin{array}{*{20}l} \log(p_{i})& = {\beta_{0}} + {\beta_{1}}\cdot\log(\text{AuM}_{i}) \\ &\quad+ {\beta_{2}}\cdot\text{A4} + {\beta_{3}}\cdot\text{A9} + {\beta_{4}}\cdot\text{A19} \\&\quad+ {\beta_{5}}\cdot\log(\text{AuM/accounts}) + {\varepsilon_{i}}.\qquad\qquad  \end{array} $$

Table 3, column 5, shows that this reestablishes the unit elasticity of AuM. We will argue below that there is a good reason to assume that the size of the accounts held at the banks contains important information about the ability of the banks to establish the tax compliance of their customers, and this affected the penalty the banks ultimately had to pay.

We also consider a quadratic specification of the size variable in Table 3, column 6, and find that it is superior to the linear specification.

Table 3, column 7, is the same specification, but using only the sample of banks for which we have accounting data (which will be used in the following regressions). The differences are small and give us no indication of important selection effects.

One might wonder if the ability of banks to pay penalties might have played a role as well. There is no mention of this in the program, but as a general principle, one purpose of criminal law is the “deterrence of further criminal conduct” (Holder (1999), Section II.B), and this can only be achieved if the fine is to some extent painful for the corporation. We therefore check whether some basic bank balance sheet data help to explain the size of the fines. First, we control for the size of the bank, measured by balance sheet or by capital, Table 3, columns 8 and 9, respectively. We find that both variables have no significant influence. Next, we measure the ability of the bank to pay by its solvency, simply measured as the share of capital in relation to the size of the balance sheet. We add plain solvency as well as its logarithmic version as regressors, Table 3, columns 10 and 11. Solvency has the expected positive coefficient and, interestingly, turns out to be significant at the 10% level.

Finally, note that it took the DoJ about 10 months from the first to the last NPA for category 2 banks. The first NPA was issued on March 30, 2015, the last on January 26, 2016. It is possible that the DoJ changed its stance as time went by. Indeed, adding the date of the NPA as a regressor produces a statistically significant negative coefficient, see Table 3, columns 12 and 13. So it appears that, controlling for everything else, the DoJ set smaller fines towards the end of the program than at the beginning. The date of the NPA is measured in years, and the variable varies from 0 for the first NPA to 0.83 for the last. The coefficient of – 1.2 therefore induces a difference of − 1.2·0.83≈−1.0 from the first to the last NPA. A logarithmic difference of this size amounts to exp(−1.0)≈1/3. This means that the fines toward the end of the program were reduced to about a third of the size of those at the beginning. It is not possible to know whether this is a reflection of an evolving policy of the DoJ, whether there were personnel changes that led to this changed behavior, or whether the DoJ chose to deal with the “hard cases” about which they suspected more wrongdoing first (without this being fully captured by our activities dummies), and hence demanded higher fines.

The last specification, Table 3, column 14, is the same as specification 13, except that we have now dropped activity A19, which is no longer significant. As a result, the solvency variable and the quadratic account size variable gain some significance, but otherwise the results are quantitatively almost unchanged.

**Fig. 2:**
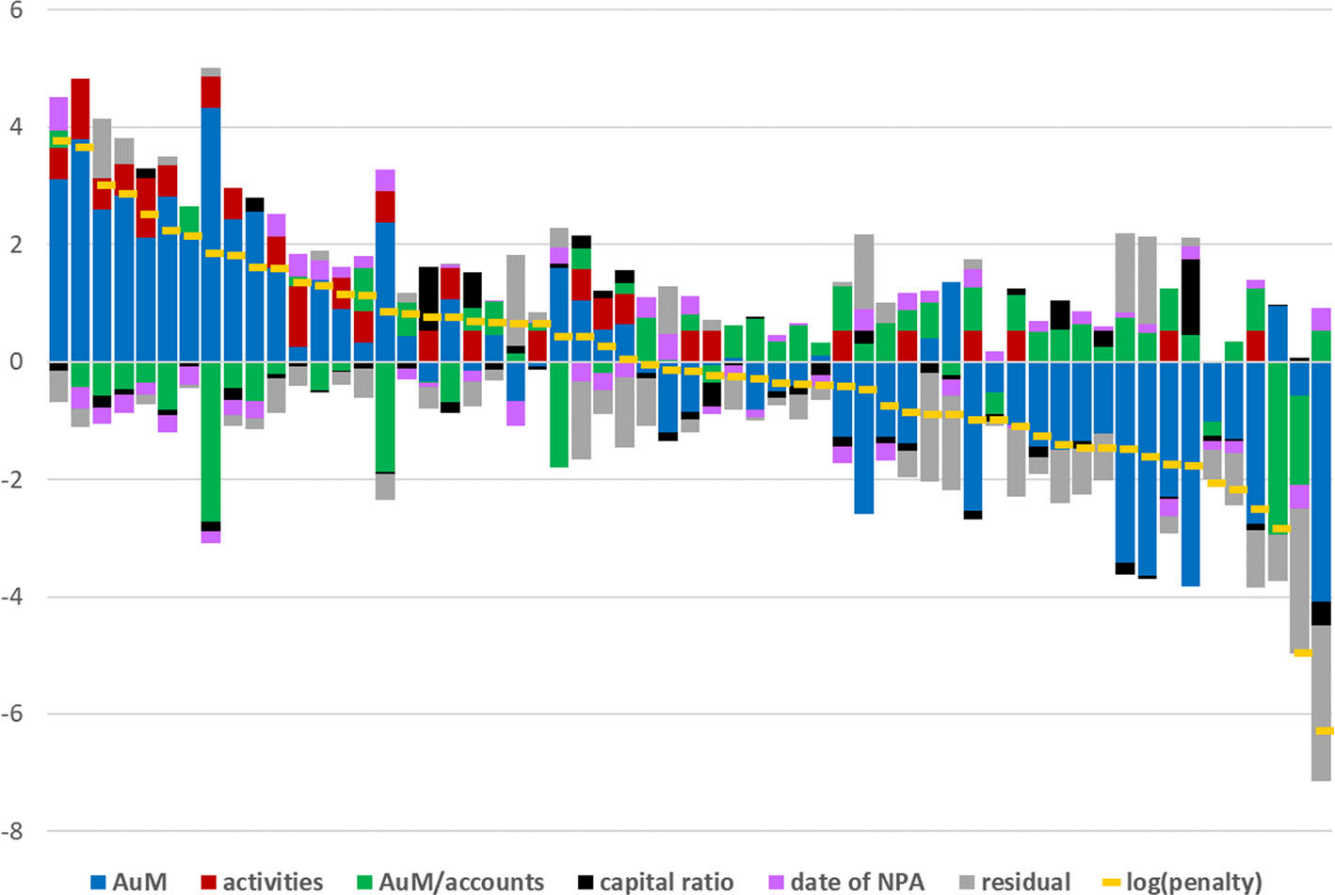
Contributions of the exogenous variables (regression version 14)

Figure 2 depicts the parts of the individual (logarithmic) penalties that are explained by the components of version (14) of the regression.^9^ AuM is clearly the dominant factor, as it should be, given the rules of the program. However, the average size of the accounts also contributes, in some instances significantly. The same applies to the two activities (A4 and A9). Solvency and the date of the NPAs appear to contribute comparatively less.

## The size variable and the role of solvency

We have found that the average size of the accounts and the solvency of the bank has a statistically significant and economically meaningful effect on the fines the banks had to pay. We now discuss possible reasons for these effects.

To understand why the average size of the accounts should have had an effect on the penalty, let us consider two banks that paid similar penalties, but had very different compositions of accounts. The (logarithmic) mean of the AuM per account across all banks in our sample is USD 0.83 million. Migrosbank reported USD 273 million U.S.-related AuM, and 898 such accounts. This makes USD 0.3 million per account on average, which is significantly smaller than the average across all banks. Migrosbank paid a fine of about USD 15 million. According to our estimate (regression (11)), if Migrosbank’s accounts had been equal to the average USD 0.83 million, its fine would have been only USD 7.3 million, which is only half of what it paid. Compare this to Rothschild Bank. This bank reported USD 1,500 million U.S.-related AuM in 332 accounts. This makes USD 4.5 million per account on average, so the account balances of Rothschild’s clients were much larger than those of the average bank. Rothschild Bank paid a smaller fine than Migrosbank, namely USD 11.5 million, despite having much more U.S.-related AuM. If it had had the average account size, its penalty would have been much larger, namely about USD 74.4 million according to our estimate.

It is possible that large clients were on average more tax compliant than small clients, or maybe it was easier for Rothschild Bank to push its larger clients into voluntary disclosure and establish the tax compliance of these accounts, while Migrosbank was not able to do so for its smaller clients. It is, unfortunately, not possible to test this hypothesis because the amount of delinquent AuM is not available.

**Fig. 3:**
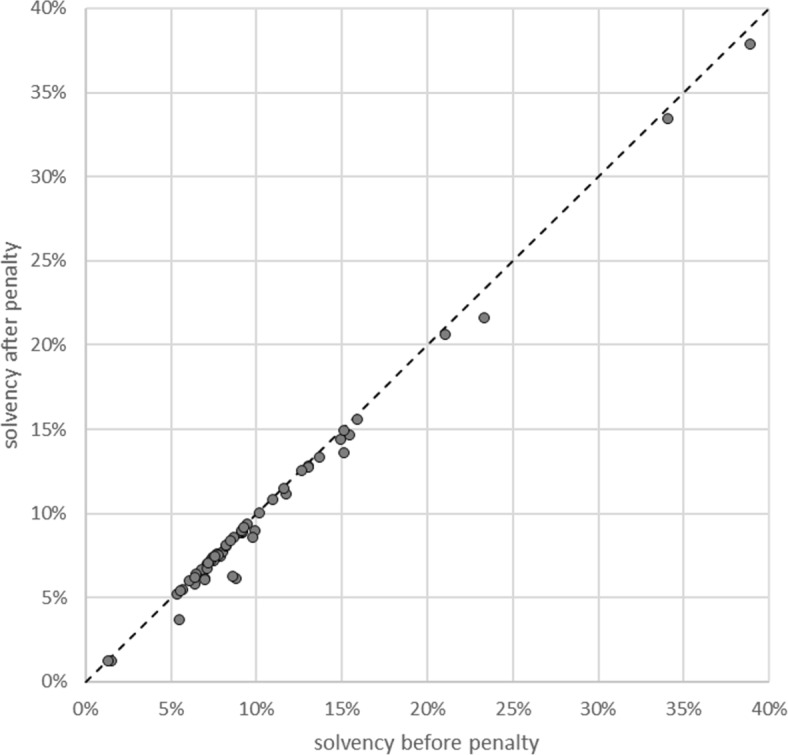
The penalties had a minimal effect on the solvency of most banks. Solvency is measured as capital divided by the size of the balance sheet. “Before penalty” means the balance sheet data of the bank as reported in the 2013 end of year annual report. “After penalty” subtracts the penalty (in CHF, using the end of year exchange rate of 1 USD = 0.8929 CHF) from capital, and divides again by the balance sheet

About the significance of solvency, one possible argument for the influence of this variable is that the DoJ wanted to avoid sending a bank into bankruptcy by charging too large a fine. On closer inspection of the data, this reasoning appears unconvincing, however. Figure 3 plots the capital ratios of the category 2 banks at the end of 2013, and the capital ratios that would have resulted if they had had to pay the fines at that moment. The fines were quantitatively much too small compared to the capital of the banks to have a pronounced effect on their solvency. The DoJ could have imposed much harsher fines without jeopardizing any of the banks. Some category 2 banks did go out of business during the program^10^, but this was not due to its becoming insolvent because of the size of the fine. The reason, then, why the solvency variable turns out to be statistically and economically significant, remains unclear.

## Conclusion

The program spells out a rule for determining the penalties. Unfortunately, it is not possible to verify that this rule was applied because the DoJ has not published the data in the necessary detail. For instance, the NPAs *do* list the maximum assets under management related to U.S. entities. However, for most banks, the DoJ does not publish *when* these accounts were in operation (which, according to the program, has an influence on the penalty), and how much of the AuM was shown by the banks to be tax compliant. Our analysis suggests that, overall, between 86 and 95% of the U.S. assets in category 2 banks were tax compliant. With the available data, we do find strong evidence that the amount of U.S.-related assets under management has a strong positive influence on the fines, as is to be expected.

The DoJ has collected and published relevant practices that banks were engaged in. We identify a small number of such activities that seem to help explain some of the variance in the fines. The activities we identify have to do with allowing customers to withdraw cash anonymously, or helping them conceal their accounts from U.S. tax authorities.

Furthermore, we find that banks with many small accounts paid larger fines than banks with few, but large, accounts. It is not completely clear why this is so. It is possible that larger clients were more tax compliant on average than smaller clients, or it is possible that the banks had more difficulty in proving tax compliance for many small clients compared to few large clients.^11^

In addition, we do find some tentative evidence that the DoJ imposed higher fines on well-capitalized banks. It is not possible to determine whether this was by design or not. It is unlikely that avoiding driving a bank into a solvency crisis was a major consideration for the DoJ; the fines were simply too small for that. However, it is possible that the DoJ felt that it could and should extract more from a well-capitalized bank. Yet, it is also possible that this statistical result stems from the necessarily miss-specified model.

Finally, we find evidence that the DoJ over the course of the program significantly reduced the fines. Banks that settled later received a better deal than banks that settled earlier. Again, it is not possible to know whether this was the intention of the DoJ, whether its policy gradually evolved over time, or whether this is connected to the sequence the DoJ set at the outset when it decided to prioritize some dossiers at the expense of others.

In conclusion, it is notable that the sums that have been transferred from the Swiss banks in categories 1 and 2 to the U.S. government are substantial. So far, these banks have paid USD 5.8 billion in fines. In addition, the legal cost for the banks must have been very high as well. There are no publicly available data on that, but a legal conflict that lasts several years for high stakes and that involves U.S. law enforcement is bound to be expensive. Moreover, U.S. tax subjects that were identified by the program were also prosecuted and were billed for taxes and fines, although no final account of these payments is publicly available.^12^ The U.S. government made a hefty gain from the program. On the other hand, the program has allowed the Swiss banking industry to leave the conflict behind in a structured and relatively quick fashion. The financial legacy that still remains today is several category 1 banks which are still awaiting a settlement.

More broadly, the program has paved the way towards the fall of Swiss bank secrecy with respect to a wide array of jurisdictions. As of today, Switzerland has signed agreements to exchange information automatically about foreign customers of Swiss banks with 41 jurisdictions. The Swiss banking industry has been permanently changed by this program.

## Abbreviations

CHF: Swiss Francs
DoJ: Department of Justice (part of United States Government)
DPA: deferred prosecution agreement
EFD: Eidgenössisches Finanzdepartement (Ministry of Public Finance of Switzerland)
EU: European Union
LOI: letter of intent
NPA: non-prosecution agreement
NTL: non target letter
OECD: Organisation for Economic Co-operation and Development
USD: US Dollars

## Footnotes

1 The law is intended to protect Swiss sovereignty and forbids official activities on Swiss soil to the benefit of foreign countries, see Husmann (2014) for a critical view of the approach that was used by the Swiss government to allow banks to cooperate.

2 The deadline for the LOI of category 3 and 4 banks was later extended to December 31, 2014, see DoJ Tax Division (2014).

3 In a DPA, charges are filed with a federal court but are dismissed after all obligations have been fulfilled. In an NPA, no charges are filed with a federal court, see Alexander and Cohen (2015).

4 The largest individual penalty for a category 2 bank was the USD 211m that was paid by BSI, a bank that has in the meantime been implicated in the 1MDB case and has been sold (https://www.finma.ch/en/news/2016/05/20160524-mm-bsi/).

5 DoJ press release number 17-910 of August 15, 2017, https://www.justice.gov/opa/pr/acting-manhattan-us-attorney-announces-agreement-swiss-asset-management-firm-resolve-criminal

6 Morgan Stanley, Goldman Sachs, Lloyds Banking Group, Banque Morval, BC Fribourg, BC Genève, Barclays Bank (Suisse), Barclays Capital (Zürich Branch of Barclays Bank London), VP Bank (Schweiz).

7 Lombard Odier, Coutts, HSZH Verwaltungs AG, Gonet, Bordier, Baumann, Banque Pasche, Standard Chartered Bank, PBZ, Finter Bank Zürich, Reichmuth, BHF-Bank, E. Gutzwiller, Société Generale Private Banking (Lugano-Svizzera), Arvest Privatbank, Leodan Privatbank, Credito Privato Commerciale, Bellerive.

8 One bank is dropped from the sample because its penalty was zero.

9 More precisely, the bars indicate the contributions for an individual bank minus the average contribution over all banks: for example, contribution_AuM_(*i*)=1.2004·[ log(AuM(*i*))−mean(log(AuM))]. For the activities dummies, we do not correct for the sample average: contribution_activities_(*i*)=0.5310·A4(*i*)+0.4996·A9(*i*).

10 Credito Privato Commerciale SA voluntarily went out of business in June 2012, but still received an NPA and paid a fine in July 2015. Banca della Svizzera Italiana (BSI) was purchased by EFG International in 2016, after losing its license in Singapore “for serious breaches of anti-money laundering requirements, poor management oversight of the bank’s operations, and gross misconduct by some of the bank’s staff” (http://www.mas.gov.sg/News-and-Publications/Media-Releases/2016/MAS-directs-BSI-Bank-to-shut-down-in-Singapore.aspx).

11 In fact, the DoJ has acknowledged the difficulty that banks faced in demonstrating tax compliance and has in the process extended the deadline for providing such evidence, see DoJ Tax Division (2014).

12 One source is a speech by former Deputy Attorney General James M. Cole, in which he mentions that, as a result of the program, over 43,000 U.S. taxpayers voluntarily disclosed to the IRS and paid over USD 6 billion in taxes and penalties (Cole 2014).
